# Mechanical Characterization of 3D-Printed Individualized Ti-Mesh (Membrane) for Alveolar Bone Defects

**DOI:** 10.1155/2019/4231872

**Published:** 2019-01-29

**Authors:** Liyun Bai, Ping Ji, Xian Li, Hui Gao, Linlin Li, Chao Wang

**Affiliations:** ^1^College of Stomatology, Chongqing Medical University, Chongqing, China; ^2^Chongqing Key Laboratory for Oral Diseases and Biomedical Sciences, Chongqing, China; ^3^Chongqing Municipal Key Laboratory of Oral Biomedical Engineering of Higher Education, Chongqing, China

## Abstract

Individualized titanium mesh holds many advantages over conventional mesh. There are few reports in the literature about the effect of mesh pore size and mesh thickness on the mechanical properties of titanium mesh. This study is designed to develop an individualized titanium mesh using computer-assisted design and additive manufacturing technology. This study will also explore the effect of different thicknesses and pore sizes of titanium mesh on its mechanical properties through 3D FEA. According to this study, the mechanical properties of titanium mesh increased when the thickness decreased (0.5 mm to 0.3 mm). With an increase in mesh diameter (3 mm to 5 mm), the mechanical properties of mesh decreased. The diameter of titanium mesh has less influence on its mechanical properties than does the thickness of the mesh. Titanium mesh with a thickness of 0.4 mm is strong enough and causes less stimulation to mucosa; therefore, it is more suitable for clinical use. In addition, parameters of titanium mesh should be decided clinically according to bone defect size, defect location, and force situation.

## 1. Introduction

Adequate bone volume in both horizontal and vertical dimensions plays a vital role in achieving long-term aesthetic and functional results in implant dentistry [1]. However, in our daily work, many patients suffer from horizontal or vertical bone deficiency, especially in cases of long lasting edentulous ridges or bone defects caused by trauma. Resorbed alveolar bone is often not sufficient to place dental implants during a prosthetic-driven procedure and frequently jeopardizes the successful outcome of an ideal implant placement. To achieve appropriate positioning of dental implants, a number of augmentation strategies have been developed to augment new bone growth, including distraction osteogenesis, block bone graft, and GBR. The process of distraction osteogenesis is complex, and compensatory bone resorption after the surgery cannot be avoided because it frequently leaves undesirable tissue scarring [2]. Autogenous onlay bone grafting is an advantageous technique for alveolar reconstruction because of its ability to provide sufficient bone volume and because of its biocompatibility. Therefore, it still remains the gold standard in reconstructive surgery. Nevertheless, the preference for this method is lessened due to the need for a donor site to harvest an autogenous bone graft, surgical complications, the need for delayed implant placement, and compensatory bone resorption after surgery [3]. GBR is one of the most utilized methods, which uses a barrier membrane to isolate the growth of soft tissue while promoting the priority of bone tissue growth [4]. The area with the bone defect is filled with an autologous bone or bone compensatory material. The bone graft material may not be close to the bone defect, and the defective anatomy and macrostructure of the biomaterial has a substantial influence on new bone formation. In order for augmentation procedures to be successful, it is imperative to stabilize the graft during healing, support the osteogenic potential of the graft materials, and close the primary soft tissue. A series of animal and clinical studies have shown that GBR can predictably facilitate bone regeneration in critical-sized bone defects [5–7]. However, it is hard to maintain a desired bone shape and volume during the entire healing period for GBR especially for large bone defects. Displacement and compression of the graft material have been cited as relevant during the postoperation period [8].

Since the introduction of Ti-mesh in 1969, titanium mesh has received close attention and has been used extensively in the reconstruction of oral and maxillofacial bone defects [9, 10]. Several advantages of titanium mesh have been suggested. Titanium mesh is rigid enough to maintain bone shape and volume, which is a basic prerequisite for any bone regeneration process. In addition, the pores of titanium mesh play a significant role both in maintaining vascular supply to a grafted defect from the overlaying periosteum and in improving tissue integrity [11]. However, manual shaping and bending, trimming, and fixation are required to apply conventional titanium mesh according to the individual defect. These processes are manually challenging, time-consuming, and highly influential with respect to the regenerative outcomes [12, 13]. Furthermore, the corners and edges of these cut and bended meshes can cause damage to the gingiva and mesh exposure site [14].

During the past decade, rapid developments have been made in CBCT technology and in additive manufacturing technology. These developments have accelerated the manufacture of complicated three-dimensional (3D) metal devices [15, 16]. Personalized titanium mesh manufactured by digital modelling and 3D printing technology can enable optimal fit between the mesh and the anatomical shape of the alveolar bone. It can also reconstruct the 3D volume and position of the bone accurately and allow the operation to be planned in advance. By avoiding manual shaping and pruning during the operation, the duration of the operation can be greatly shortened.

An important criterion for the success of titanium mesh seems to be its mechanical strength that can withstand the desired load and space maintenance which is affected by its thickness and pore size properties. However, the stiffness of Ti-mesh may cause irritation to mucosa, resulting in membrane exposure. Accordingly, a proper thickness and pore size must be balanced with the likelihood of irritation when using Ti-mesh. The usual available thicknesses of titanium mesh range from 0.1 to 0.6 mm and have no uniform specifications, neither does the pore size [17–23]. The thickness and pore size of titanium mesh could influence the amount of new bone generated beneath the mesh; therefore, careful selection of the appropriate pore size and thickness can play a critical role in isolating the growth of soft tissue and promoting bone tissue growth. The purpose of this study was to design an individualized titanium mesh and to explore the effect of titanium mesh with different thicknesses and pore sizes on its mechanical properties. In the present study, we have designed and manufactured a customized titanium mesh based on computerized tomography (CT) of individual patients. The designed titanium mesh with different diameters and thicknesses were explored based on their mechanical strength through a three-point bending test and FEA.

## 2. Materials and Methods

### 2.1. Three-Point Bending Tests

#### 2.1.1. Preparation of Test Specimen

**Figure 1:**
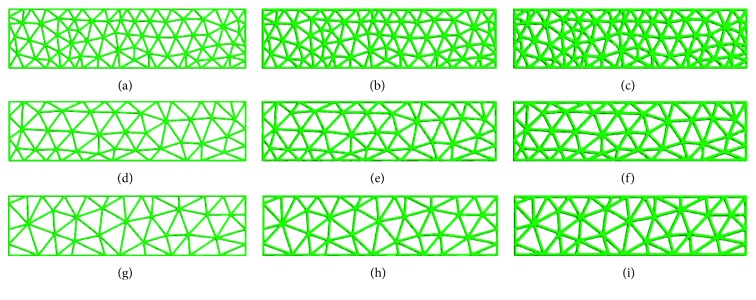
Model of standard specimens with different thicknesses and diameters in 3-Matic: (a) thickness: 0.3 mm, diameter: 3 mm; (b) thickness: 0.4 mm, diameter: 3 mm; (c) thickness: 0.5 mm, diameter: 3 mm; (d) thickness: 0.3 mm, diameter: 4 mm; (e) thickness: 0.4 mm, diameter: 4 mm; (f) thickness: 0.5 mm, diameter: 4 mm; (g) thickness: 0.3 mm, diameter: 5 mm; (h) thickness: 0.4 mm, diameter: 5 mm; (i) thickness: 0.5 mm, diameter: 5 mm.

**Figure 2:**
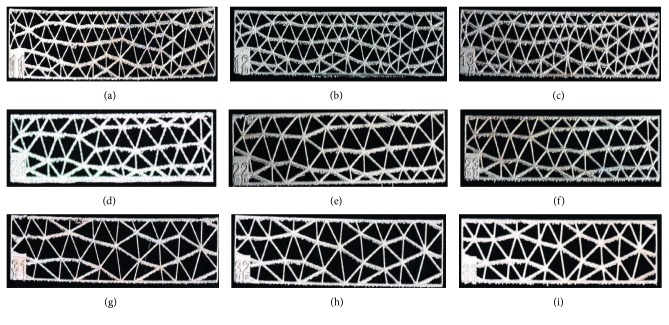
SLM product of standard specimens with different thicknesses and diameters: (a) thickness: 0.3 mm, diameter: 3 mm; (b) thickness: 0.4 mm, diameter: 3 mm; (c) thickness: 0.5 mm, diameter: 3 mm; (d) thickness: 0.3 mm, diameter: 4 mm; (e) thickness: 0.4 mm, diameter: 4 mm; (f) thickness: 0.5 mm, diameter: 4 mm; (g) thickness: 0.3 mm, diameter: 5 mm; (h) thickness: 0.4 mm, diameter: 5 mm; (i) thickness: 0.5 mm, diameter: 5 mm.

Nine groups of standard titanium mesh specimens with diameters of 3.0 mm, 4.0 mm, or 5.0 mm and thicknesses of 0.3 mm, 0.4 mm, or 0.5 mm were designed using 3-Matic software (Figure 1); the length of the sample was 40 mm, and the width was 10 mm. The data were converted to a stereolithography (STL) file and were transferred to an SLM-RP molding machine LaserCUSING® (Concept Laser GmbH, Germany). Finally, the SLM products were obtained (Figure 2).

#### 2.1.2. Standard Specimens Mechanical Testing

The mechanical strength of titanium mesh was analysed using three-point bending tests on standard specimens. After the etching treatment and the polishing procedure, bending strength was tested using GB/T14452-93. Nine groups of specimens were fabricated with five specimens printed for each group (Figure 2). The three-point bending tests were done on a universal mechanical tester (C43.104.MTSLtd., China). The strided distance (*L*_s_) was 10 mm. The compressive load was vertically applied at a rate of 1 mm/min. Constant pressure was applied until the specimen was destroyed. The peak force value of the bending or breaking of each specimen was recorded. The load displacement curve was generated, and the bending strength was calculated. The results were shown as the mean ± standard deviation. Data were analysed statistically with analysis of variance (one-way ANOVA) (SPSS 13) followed by a Tukey's post hoc analysis using a significance level of *p* < 0.05.

### 2.2. Customized Design for Titanium Mesh

**Figure 3:**
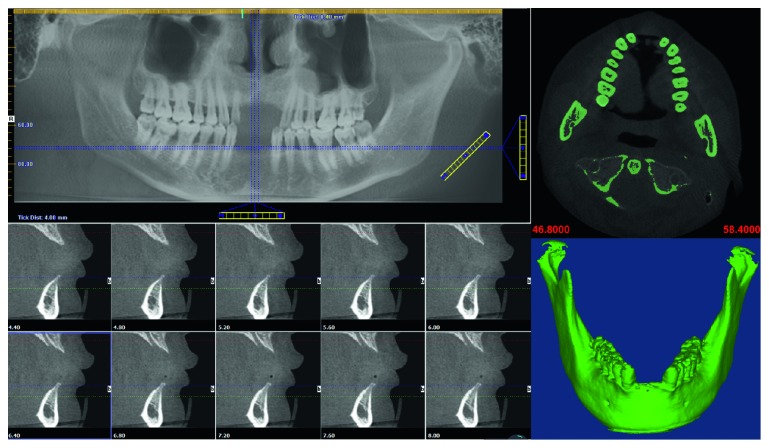
Preoperative CBCT scan and the 3D model of the patient's mandible.

**Figure 4:**
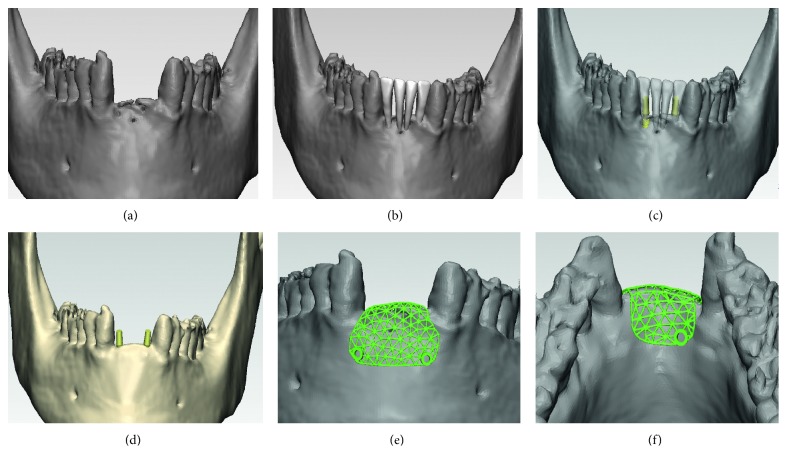
Steps involved in customized titanium mesh design using 3-Matic® and Geomagic Studio®: (a) mandible model; (b) arranged missing teeth; (c) arranged dental implant; (d) virtual bone augmentation; (e, f) models of titanium mesh.

**Figure 5:**
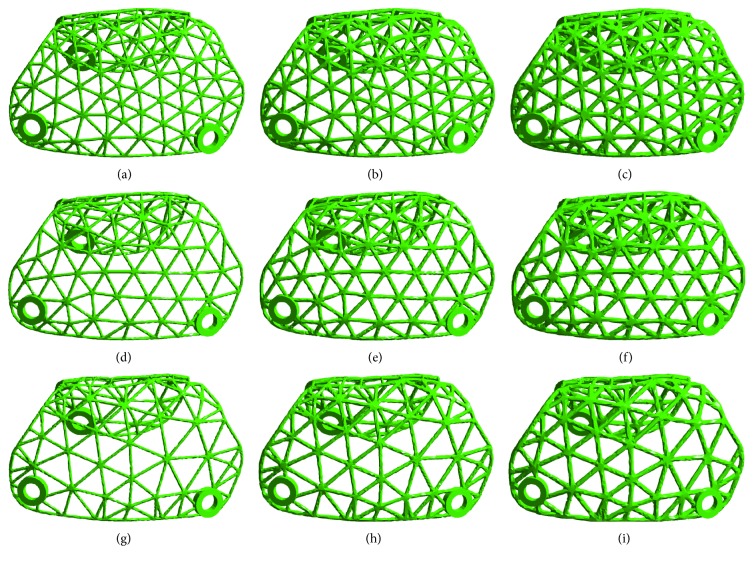
Individual titanium mesh with different thicknesses and diameters: (a) thickness: 0.3 mm, diameter: 3 mm; (b) thickness: 0.4 mm, diameter: 3 mm; (c) thickness: 0.5 mm, diameter: 3 mm; (d) thickness: 0.3 mm, diameter: 4 mm; (e) thickness: 0.4 mm, diameter: 4 mm; (f) thickness: 0.5 mm, diameter: 4 mm; (g) thickness: 0.3 mm, diameter: 5 mm; (h) thickness: 0.4 mm, diameter: 5 mm; (i) thickness: 0.5 mm, diameter: 5 mm.

Cone-beam computerized tomography (CBCT) scans were taken of a patient, with the patient's approval. The patient had anterior teeth missing due to trauma, leaving a large bony defect. Based on Digital Imaging and Communications in Medicine (DICOM) data of the CBCT scan, a model of the patient's anatomy was established. The software package Mimics 17.0 (Materialise, Leuven, Belgium) was utilized to convert the slice data into a 3D model of the patient's bone tissue, using the system's built-in threshold function. The 3D model of the patient's mandible anatomy generated in the Mimics® software is demonstrated in Figure 3. The 3D model created in Mimics was saved as an STL (standard tessellation language) file for further processing of the mandible and to construct the titanium mesh model (Figure 4(a)). Steps involved in customized titanium mesh design were as follows: First, the missing teeth were arranged based on the patient's adjacent and opposite teeth (Figure 4(b)). Second, to determine the required bone volume (horizontally and vertically), the virtual surgical procedure placed the implants in the atrophic edentulous area. Two implants (diameter of 3.4 mm and length of 10 mm) were arranged on the basis of the planned position of the missing teeth where insufficient bone was available (Figure 4(c)). Third, after the virtual positioning of the dental implant, virtual bone augmentation (horizontally and vertically) guided by the three-dimensional ideal location of the implant was taken (Figure 4(d)). To inhibit the biological formation of pseudoperiosteum, an overcontouring of 1.5 mm of bone volume was devised in order to cover the titanium mesh. Finally, the titanium mesh was designed using Geomagic Studio software (Geomagic Company, NC, USA) and the 3-Matic software (Figures 4(e) and 4(f)). The shape of the mesh weave was made as a triangle in order to increase the contact area between the titanium mesh and the bone tissue. To remove the possibility of creating a pathway for the penetration of bacteria, the mesiodistal end of the titanium mesh was separated from the adjacent tooth by at least 2 mm. In addition, the edge of the mesh was designed as smoothly as possible to avoid postoperative mucosal exposure due to the tension of the flap. The screw holes for fixing the device were also designed in the same manner. The diameter of the holes was set to 1.7 mm. Two holes on the buccal side and one on the lingual side were made for inserting the miniscrews with which to affix the titanium mesh to the underlying cortical bone and cancellous bone. Nine groups of individualized titanium mesh with diameters of 3.0 mm, 4.0 mm, or 5.0 mm and thicknesses of 0.3 mm, 0.4 mm, or 0.5 mm were designed using 3-Matic software and Geomagic Studio software in the same manner (Figure 5).

### 2.3. Standard Specimens FEA

**Figure 6:**
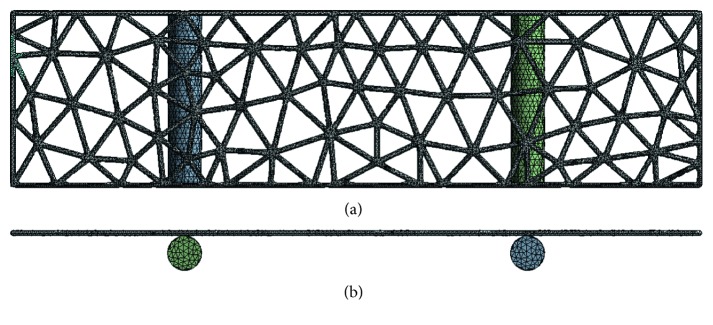
3D FE models of standard specimens: (a) back view; (b) top view.

The 3D geometries of the standard specimens were imputed to ANSYS software to simulate the three-point bending test. Nine groups of 3D FE models of standard titanium mesh of different diameters and thicknesses were built (Figure 6). The applied force was 30 N in an axial direction. The data regarding stress, strain, and displacement of the titanium mesh were outputted for further analysis.

### 2.4. Individual Titanium Mesh FEA

#### 2.4.1. Model Design

**Figure 7:**
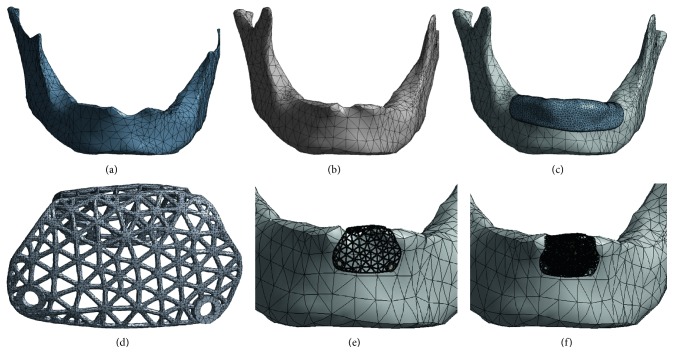
3D FE models of individual titanium mesh: (a) cancellous bone model; (b) cortical bone model; (c) mucosa model; (d) titanium mesh model; (e) mesh detail containing the titanium mesh, cortical bone, and cancellous bone; (f) mesh detail containing the titanium mesh, bone graft, cortical bone, and cancellous bone.

The 3D geometries of the mandible including the cortical bone and the cancellous bone were modeled using Geomagic Studio software. The bone graft material that filled in bone defects was generated using 3-Matic software. The mucosa covering the mandible with an average thickness of 2 mm was also generated using Geomagic Studio software. The titanium screws used to affix the titanium mesh to the underlying cortical bone were designed by SolidWorks® 12.0 (SolidWorks Corporation, Velizy-Villacoublay, France). The model of titanium mesh and bone graft was meshed in 3-Matic and formatted with a .cdb file for further processing. The geometries of the cortical bone, cancellous bone of mandible, mucosa, and titanium screws were then calculated using the FE software ANSYS (Swanson Analysis System Co., Houston, TX, USA). Nine groups of 3D FE models of individualized titanium mesh with different diameters and thicknesses were built (Figure 7). The total number of elements and nodes is listed in Table 1. The titanium mesh and titanium screws were made of Ti6Al4V titanium alloy. The material properties of cortical bone, cancellous bone, mucosa, soft callus, and titanium alloy were taken from the literature and are listed in Table 2 [24–26].

#### 2.4.2. Contact Management and Loading Conditions

To simulate the intraoral situation precisely, the cortical bone was bonded to the cancellous bone, and the mucosa and the bone were considered fixed together. The titanium mesh was affixed to the underlying cortical bone using titanium screws, and the interface between bone and titanium screws was presumed fixed. However, the interface between titanium mesh and screws was not presumed fixed, nor was the junction of titanium mesh and cortical bone, in order to simulate the early stage of healing. The mandibles were constrained by the temporomandibular joint (TMJ) and masticatory muscles. These muscles include the temporalis and masseter muscle attachments at the lateral surface, and attachments for the digastric, temporalis, lateral pterygoid, and medial pterygoid muscles on the medial surface [24]. A functional loading force of 100 N in an axial direction was applied. The data regarding stress and displacement of the titanium mesh were outputted for further analysis.

### 2.5. Fabrication of Designed Individualized Titanium Mesh

**Figure 8:**
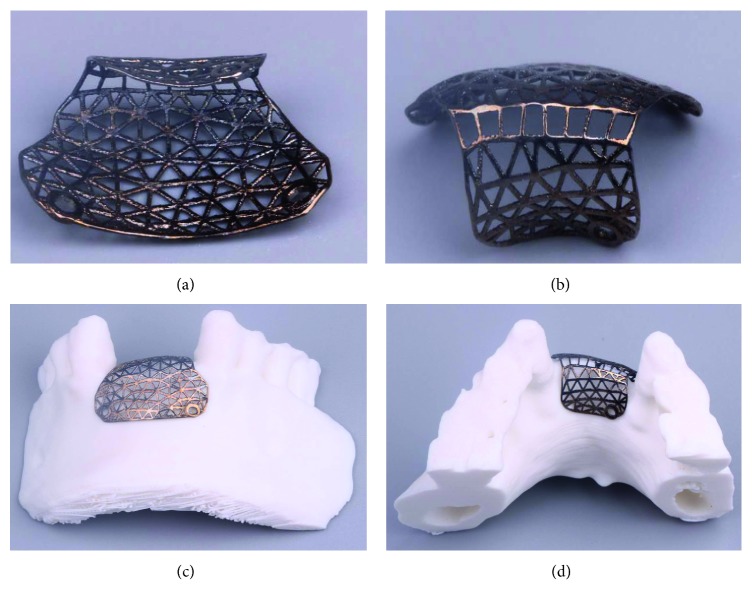
SLM product of individual titanium mesh.

The data regarding customized mesh in a stereolithography (STL) file for bone augmentation were directly fabricated using a LaserCUSING® (Concept Laser GmbH, Germany). Finally, the SLM products were obtained (Figure 8).

## 3. Results

### 3.1. Three-Point Bending Test

**Figure 9:**
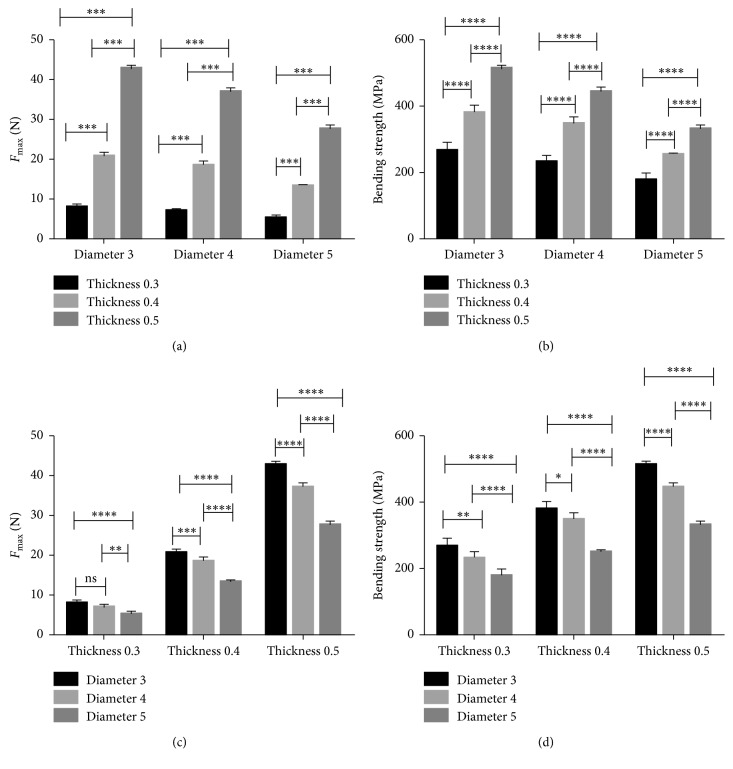
(a, c) The maximum force values and (b, d) bending strength values in standard specimens (one-way ANOVA with Tukey's post hoc analysis, *p* < 0.05).

The peak force value (N) and three-point bending strength (MPa) of the standard specimens are shown in Table 3. As the thickness of the titanium mesh increased, the maximum force value and the bending strength of the titanium mesh increased. When the diameter increased, the maximum force value and bending strength of titanium mesh decreased (Figure 9). The bending strength of the 0.5 mm group was highest, and the 0.3 mm group was lowest. There was a significant difference in bending strength among the different mesh thickness groups (*p* < 0.05). The bending strength of the 3 mm group was highest, and the 5 mm group was lowest. There was also a significant difference in bending strength among the different mesh diameter groups (*p* < 0.05).

### 3.2. Standard Specimens FEA

**Figure 10:**
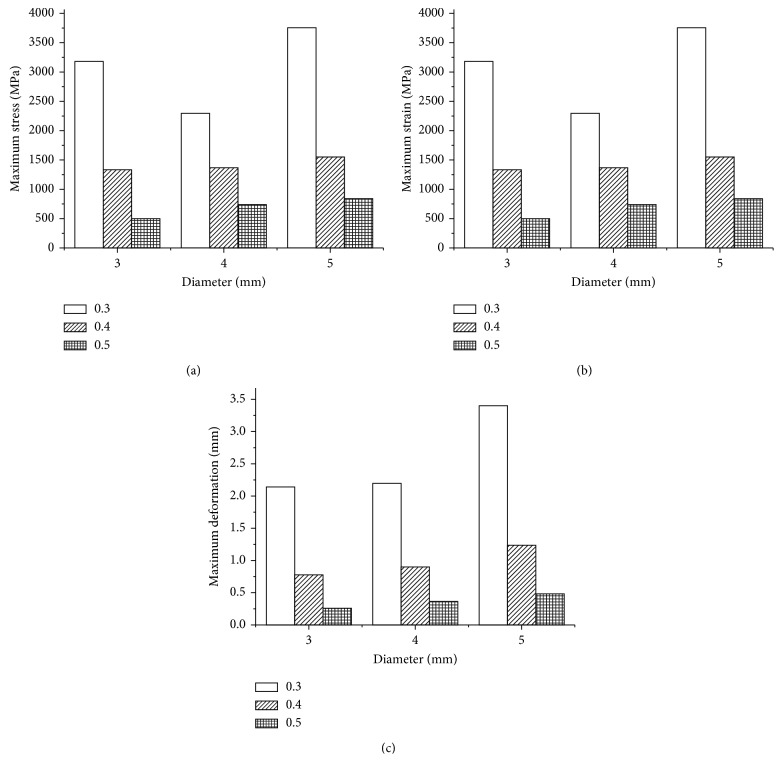
(a) Maximum stress values, (b) maximum strain values, and (c) maximum deformation values in standard specimens.

With continual application of a force of 30 N, the maximum values of the three evaluated parameters (deformation and von Mises stress and strain) of the standard specimens were compared within the different diameter and thickness settings. The maximum stress value (in MPa), strain value, and deformation value in each model are displayed in Figure 10. Among the nine models evaluated in this study, the maximum stress value, deformation value, and the maximum strain value were observed in specimens 5 mm in diameter and 0.3 mm in thickness. The minimum stress value, deformation value, and the minimum strain value were observed in specimens 3 mm in diameter and 0.5 mm in thickness. The results showed that the maximum value of stress and strain as well as the deformation distributions had increased when the thickness decreased (0.5 to 0.3 mm). In addition, the level of stress, strain, and the deformation distributions increased with the increase in mesh diameter (3 to 5 mm).

### 3.3. Customized Titanium Mesh FEA

**Figure 11:**
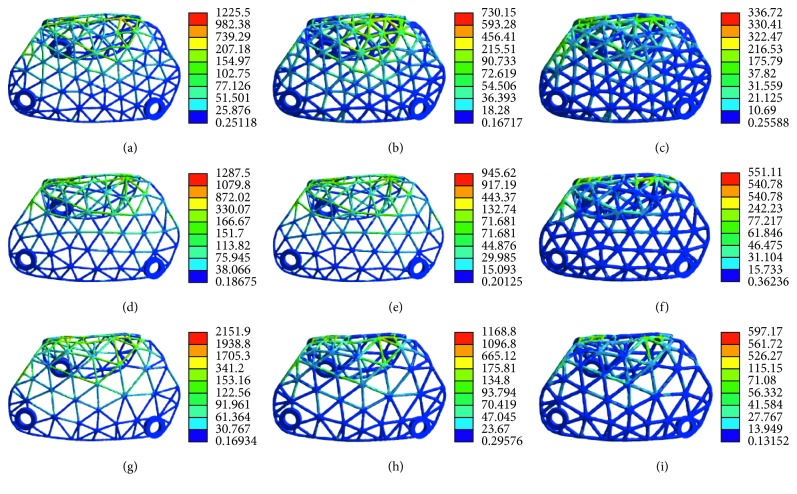
The von Mises stress analysis of customized titanium mesh with different diameters and thicknesses: (a) thickness: 0.3 mm, diameter: 3 mm; (b) thickness: 0.4 mm, diameter: 3 mm; (c) thickness: 0.5 mm, diameter: 3 mm; (d) thickness: 0.3 mm, diameter: 4 mm; (e) thickness: 0.4 mm, diameter: 4 mm; (f) thickness: 0.5 mm, diameter: 4 mm; (g) thickness: 0.3 mm, diameter: 5 mm; (h) thickness: 0.4 mm, diameter: 5 mm; (i) thickness: 0.5 mm, diameter: 5 mm.

**Figure 12:**
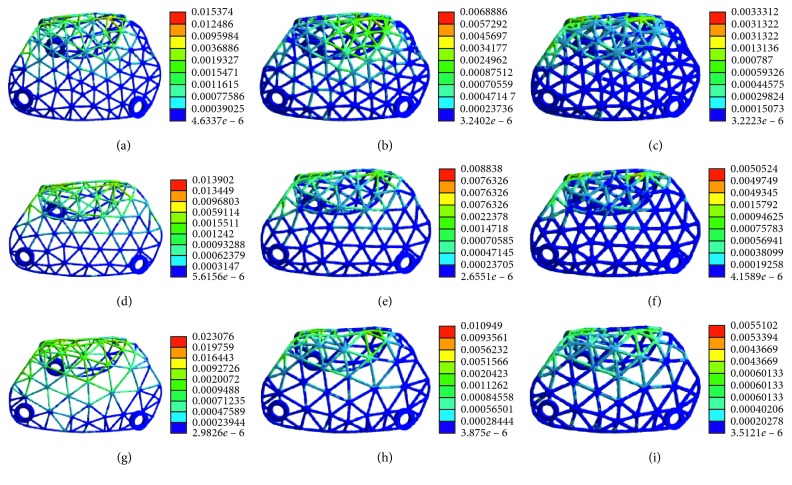
Strain distribution of customized titanium mesh with different diameters and thicknesses: (a) thickness: 0.3 mm, diameter: 3 mm; (b) thickness: 0.4 mm, diameter: 3 mm; (c) thickness: 0.5 mm, diameter: 3 mm; (d) thickness: 0.3 mm, diameter: 4 mm; (e) thickness: 0.4 mm, diameter: 4 mm; (f) thickness: 0.5 mm, diameter: 4 mm; (g) thickness: 0.3 mm, diameter: 5 mm; (h) thickness: 0.4 mm, diameter: 5 mm; (i) thickness: 0.5 mm, diameter: 5 mm.

**Figure 13:**
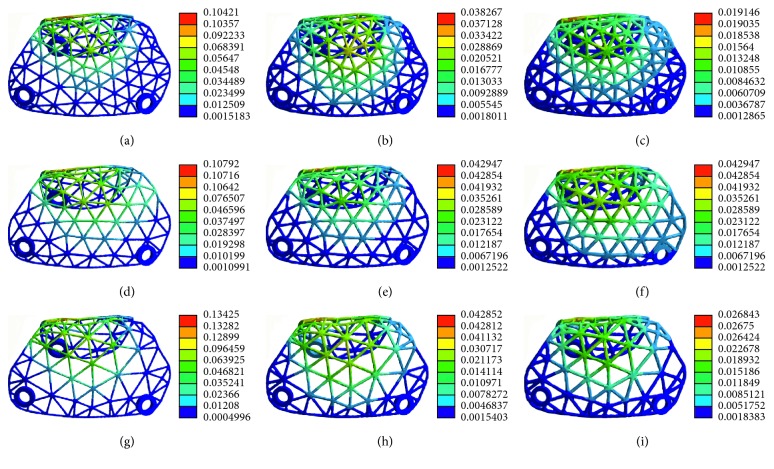
Deformation distribution of customized titanium mesh with different diameters and thicknesses: (a) thickness: 0.3 mm, diameter: 3 mm; (b) thickness: 0.4 mm, diameter: 3 mm; (c) thickness: 0.5 mm, diameter: 3 mm; (d) thickness: 0.3 mm, diameter: 4 mm; (e) thickness: 0.4 mm, diameter: 4 mm; (f) thickness: 0.5 mm, diameter: 4 mm; (g) thickness: 0.3 mm, diameter: 5 mm; (h) thickness: 0.4 mm, diameter: 5 mm; (i) thickness: 0.5 mm, diameter: 5 mm.

**Figure 14:**
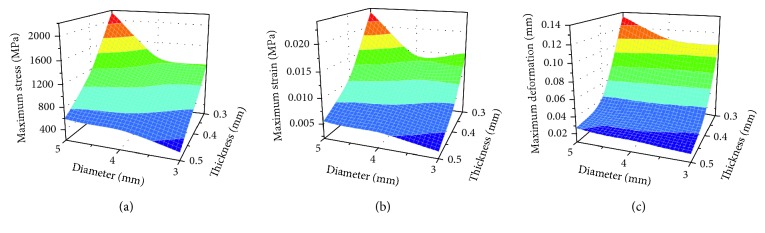
Response graphs of (a) maximum stress, (b) maximum strain, and (c) maximum deformation values of individual titanium mesh.

The pattern of stress distribution was similar for all models (Figure 11). The von Mises criteria showed stress concentration of the titanium mesh on the crest of the alveolar ridge and at the labial side near the top of the alveolar ridge. However, the increase of mesh thickness generated less concentrated stress, and the maximum stress level decreased. All models presented a similar pattern of strain distribution with contour lines of different colours representing mesh strain (Figure 12). The results showed that the high strain value increased when the thickness decreased. Furthermore, the high level of strain increased with the increase in mesh diameter. As shown in Figure 13, the displacement distribution patterns are shown as contour lines of different colours to represent mesh displacement with the bite force of 100 N. The increase of the mesh diameter (3 to 5 mm) tends to increase the displacement tendency. However, the increase of mesh thickness (0.3 to 0.5 mm) tends to decrease the displacement tendency. Among the nine models evaluated in this study, the maximum stress value and the maximum strain value were observed in specimens 5 mm in diameter and 0.3 mm in thickness. The minimum stress value and the minimum strain value were observed in specimens 3 mm in diameter and 0.5 mm in thickness. Compared with change in thickness, the stress, strain, and displacement of titanium mesh did not increase much with the change of the pore size of the mesh (Figure 14).

## 4. Discussion

The current approach presented an innovative multidisciplinary protocol for designing individualized titanium mesh based on prosthetically guided bone regeneration. We have designed a customized titanium mesh from a patient's CBCT scan dependent on the desired implant positions. The four main advantages of this system were as follows. (i) Extensive indications: current commercial flexible titanium meshes need to be fixed by implant and cannot be applied in cases of severe bone deficiency. The customized titanium could be used in various types of bone defects, especially those with large bone deficiency. (ii) The individualized titanium mesh fitted securely to the morphology of the alveolar bone, so the surgeon did not need to trim or bend the mesh during the operation. (iii) The individualized titanium mesh designed on the desired implant position allowed a definite size and volume of augmentation. This could minimize the required amount of harvested or synthetic graft material. (iv) Round and blunt edges of the customized titanium mesh were developed to prevent mucosal irritation, resulting in a reduction of mesh exposure after operation.

The study explored the effect of different thicknesses and pore sizes of titanium mesh on its mechanical properties. Following surgery, numerous types of compressions may arise if the titanium mesh does not work as a stiff box, potentially caused both by the impact of an unexpected food block and muscle movement. The deformation of titanium mesh during the healing period can cause detrimental and unfavourable variations in the planned augmented bone, thus influencing the overall augmented bone quality and volume. Therefore, the titanium mesh must be stiff enough to sustain pressure from the overlying flaps, muscle movement, or mastication loading until the blood clot underneath the membrane has matured enough to support it [12].

Regarding the optimal thickness of the titanium mesh, the three-point bending test and the FEA results showed that the mechanical properties were enhanced when the thickness increased, whether it was a standard sample or personalized titanium mesh. The FEA results of individualized titanium mesh indicate that the largest von Mises stress value of titanium mesh with a thickness of 0.3 mm is over the yield strength of titanium alloy (*σ* = 780–950 MPa) [27], suggesting that the titanium mesh 0.3 mm in thickness may be fractured and is not safe for bone augmentation. The FEA results of individualized titanium mesh indicate that the maximum stress on titanium mesh with the thicknesses of 0.5 and 0.4 mm are under the yield strength of titanium alloy (*σ* = 780–950 MPa) [27], suggesting that titanium meshes 0.5 and 0.4 mm in thickness are safe for bone augmentation. The thinner titanium mesh may result in less mucosal irritation which can lead to exposure of the mesh. Therefore, the appropriate thickness of a titanium mesh must be rigid enough to maintain space for bone regeneration and should be balanced with the likelihood of irritation. The thickness should not adversely influence its ability to integrate with the surrounding tissue when using titanium mesh for GBR [28]. Titanium mesh with a thickness of 0.4 mm can not only bear enough strength but also lead to less stimulation of mucosa; therefore, it is more suitable for clinical use.

Membrane pores were considered to play an essential role in maintaining blood supply and in facilitating bone regeneration and soft tissue healing [29]. However, in conventional titanium mesh, these multiporous properties created sharp edges when the material was manually shaped or bent and may offer a simple pathway for microbial invasion into the healing site [30]. The effect of pore size on the osteogenesis of titanium mesh is controversial. One study advocated that macroporous (in the millimetre range) membranes promoted greater bone regeneration and prevented significant soft tissue ingrowth compared with microporous membranes [22]. Another study indicated that microporous (in the micron range) titanium mesh had some potential for greater bone regeneration [19]. The three-point bending test and the FEA results demonstrated that the mechanical properties weakened with the increase in mesh diameter, whether it was a standard sample or individualized titanium mesh. In the range of 3–5 mm, the diameter of titanium mesh has less influence on its mechanical properties. Further animal or clinical research should be directed towards identifying the mesh pore size that would inhibit excessive soft tissue ingrowth and facilitate bone ingrowth in ridge augmentation procedures.

For the anterior teeth with a single tooth missing, a titanium mesh with a thickness of 0.3 mm could be chosen and patients should be told not to bite hard objects. For the patients with thin mucosa, in order to reduce the exposure of the titanium mesh, a thin titanium mesh should be selected. For multiple tooth defects, especially in the posterior teeth, a thicker titanium mesh should be chosen to prevent the titanium mesh from breaking. Therefore, parameters of titanium mesh should be decided clinically according to defect size, defect location, and force situation.

## 5. Conclusion

The stiffness of Ti-mesh can maintain space better than other membrane and is conducive to the shaping of bone-grafting materials but may result in mucosal irritation that leads to exposure of the membrane. The custom-made titanium mesh offers greater advantages over commercial meshes by shortening the duration of surgery and eliminating the risk of postoperative infections. Within the limitations of this research regarding the optimal thickness of the titanium mesh, the results indicate that the titanium mesh with a thickness of 0.4 mm is more suitable for clinical use. Regarding the optimal pore size of the titanium mesh, the diameter of titanium mesh had less influence on its mechanical properties. Further animal or clinical research should be directed towards identifying an optimal mesh pore size that would inhibit excessive soft tissue ingrowth and facilitate bone ingrowth in ridge augmentation procedures. Otherwise, the parameters of titanium mesh should be decided clinically according to defect size, defect location, and force situation.

## Figures and Tables

**Table 1:** Number of elements and nodes.

Materials	Elements	Nodes
Cortical bone	80044	18680
Trabecular bone	47076	11721
Titanium mesh	36757	11994
Titanium screws	41298	8609
Graft bone	218362	65596
Mucosa	28204	8527

**Table 2:** Material properties.

Materials	Young's modulus (MPa)	Poisson's ratio
Cortical bone	15000	0.3
Trabecular bone	1500	0.3
Titanium alloy	110000	0.3
Soft callus	0.2	0.167
Mucosa	1	0.37

**Table 3:** The maximum force value (N) and three-point bending strength (MPa) (*n*=5, *x* ± *s*).

Diameter (mm)	Thickness (mm)	Maximum force value (N)	Bending strength (MPa)
3	0.3	8.00 ± 0.71	266.83 ± 23.59
3	0.4	20.74 ± 0.85	380.01 ± 21.24
3	0.5	42.71 ± 0.81	512.54 ± 9.66
4	0.3	6.97 ± 0.56	232.26 ± 18.58
4	0.4	18.53 ± 1.03	347.50 ± 19.33
4	0.5	37.03 ± 1.08	444.22 ± 12.98
5	0.3	5.26 ± 0.74	178.00 ± 20.99
5	0.4	13.37 ± 0.26	250.77 ± 4.81
5	0.5	7.58 ± 0.91	330.94 ± 10.88

## Data Availability

All data included in this study are available upon request by contacting the corresponding author.
